# Melioidosis in New Caledonia

**DOI:** 10.3201/eid1110.050823

**Published:** 2005-10

**Authors:** Simon Le Hello, Bart J. Currie, Daniel Godoy, Brian G. Spratt, Marc Mikulski, Flore Lacassin, Benoit Garin

**Affiliations:** * Institut Pasteur de Nouvelle-Calédonie, Nouméa, New Caledonia; † Menzies School of Health Research, Charles Darwin University, Northern Territory, Australia; ‡ St Mary's Hospital, London, United Kingdom; § Gaston Bourret Hospital of Nouméa, Nouméa, New Caledonia

**Keywords:** Melioidosis, New Caledonia, MLST, dispatch

## Abstract

Recognized melioidosis-endemic areas are widening. In the South Pacific, melioidosis is endemic in New Caledonia, northern Australia, and Papua New Guinea. We report the first 4 documented cases of human melioidosis from New Caledonia. Molecular typing of 2 *Burkholderia pseudomallei* isolates suggests a link to Australian strains.

Melioidosis, a tropical disease endemic in areas of Southeast Asia and northern Australia (*1*), is caused by *Burkholderia pseudomallei*, an environmental bacterium that lives in soil and surface water (*2*). This disease is increasingly recognized as an emerging problem because it is more often recognized and identified. Recent new "hot spots" have been reported in Mauritius (*3*), the Indian subcontinent (*4*), the Americas (*5**,**6*), and the Caribbean (*7*). In the South Pacific, melioidosis is a rare (*8*), but likely underdiagnosed, illness.

New Caledonia is located in Oceania, ≈2,000 km northeast of Sydney, Australia. With nearly 220,000 inhabitants, the country is a discrete epidemiologic entity with Pacific Island characteristics and a multicultural (mainly Melanesian, European, and Polynesian) population. The country has 2 provinces on the main island and a third province of smaller islands. Total land area is 18,575 km^2^, slightly smaller than New Jersey. In New Caledonia, 4 cases of melioidosis have been diagnosed in the last 6 years. All were in Melanesians living in the Northern Province, none of whom had traveled abroad, which suggests this area is another discrete focus of endemic melioidosis. The average age of patients was 53 years; 3 had recognized risk factors for melioidosis, and all 4 were heavy kava drinkers.

## The Cases

Melioidosis was confirmed for the first time in New Caledonia in February 1999 in a 46-year-old male nurse who worked at the health center of Ouegoa, a village in the Northern Province. He was admitted to the hospital with fever, acute renal insufficiency, pneumonia, and septic shock. Blood culture grew an oxidase-positive, gram-negative rod, which was initially identified as a *Pseudomonas* sp. by the local laboratory but later confirmed as *B. pseudomallei* by the Pasteur Institute when the patient was transferred to the hospital in the capital city, Nouméa. He had been treated for tuberculosis 20 years earlier. He required intubation and ventilation, and treatment was begun with a combination of ceftazidime and amikacin. Ten days after admission, when *B. pseudomallei* infection was confirmed, treatment was changed to the combination of imipenem and trimethoprim/sulfamethoxazole. Numerous cutaneous abscesses cultured *B. pseudomallei*, and he slowly recovered.

The second case came from the same location of Ouegoa but not from the same tribe. The patient was a 43-year-old man with diabetes mellitus and chronic renal failure who required a kidney transplant in 2001. He was taking the immunosuppressant medication tacrolimus when "*B. gladioli*" septicemia was diagnosed in April 2002. Two months later, he was admitted to the hospital with pneumonia, and blood cultures grew *B. pseudomallei*. He made a full recovery after therapy with a combination of ceftazidime and trimethoprim/sulfamethoxazole.

In July 2003, a 58-year-old man, living in Poum, ≈50 km from Ouegoa, was admitted with fever, pneumonia, and renal impairment. This man had tuberculosis in 1993 and was a smoker and alcoholic. Treatment was with amoxicillin/clavulanate for 24 hours followed by a combination of cefotaxime, ofloxacin, and metronidazole for 48 hours. He was transferred to Nouméa with severe hypoxemia from bilateral pneumonia and hepatocellular insufficiency. A gram-negative bacillus was isolated from bronchial secretions. Therapy was changed to a combination of ceftazidime and ciprofloxacin, and he slowly improved. The organism was identified as *B. pseudomallei* 7 days after the patient's transfer to Nouméa. A lung lobectomy was performed after 2 months for persisting pulmonary infection, and *B. pseudomallei* was cultured from the surgical specimen. The patient continued to receive trimethoprim/sulfamethoxazole on a long-term basis.

In June 2004, a 67-year-old woman with a history of diabetes mellitus came to the hospital with abscesses on her thigh, from which *B. pseudomallei* was cultured. She was otherwise healthy and recovered fully after receiving therapy for melioidosis. She lives in Poindimie, about 200 km southeast of Ouegoa.

The bacterium, also called Whitmore bacillus, is an oxidase-positive, gram-negative rod. It is commonly misidentified, sometimes as various species of the *Pseudomonas* genus. The bacterium grows aerobically on most agar media and usually produces clearly visible colonies within 24 hours at 37°C. In our cases, *B. pseudomallei* was identified with API 20NE and API 32GN (API system SA, Lyon, France). Two of the isolates were sent to the Centre d'Identification Moléculaire des Bactéries of Pasteur Institute in Paris, where the identity was confirmed by amplifying and sequencing the 16S rRNA gene. These strains were also defined by multilocus sequence typing (MLST) at Imperial College, London, and compared with isolates from Australia and Thailand. Both strains were new sequence types. When a minimum evolution analysis was performed on the concatenated sequences of the 2 strains and compared with Australian and Thai strains on the MLST website (http://bpseudomallei.mlst.net/), the New Caledonian strains clustered well within groups of Australian sequence types (Figure). Furthermore, 1 of the strains was a single-locus variant (i.e., 6 of 7 alleles identical) of a strain from the east coast of Australia. This comparison suggests that New Caledonian *B. pseudomallei* strains are linked to Australian strains (*9*). New Caledonia is a fragment of the ancient continent of Gondwana and subsequently separated from Australia and New Zealand. The diverse but distinct phylogeny of strains of *B. pseudomallei* in Southeast Asia and Australia may reflect geographic isolation over long periods.

**Figure:**
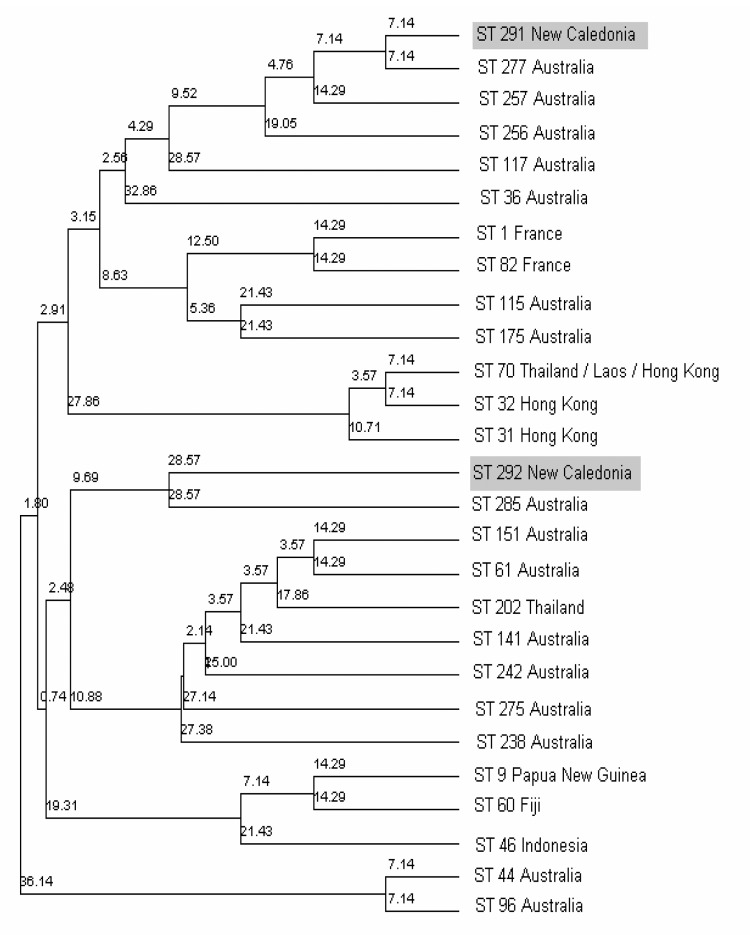
Phylogenetic tree constructed from the concatenated sequences of the 7 multilocus sequence type loci from Burkholderia pseudomallei isolates, illustrating the relationship of the 2 Caledonian strains to Australian and Thai isolates.

## Conclusions

In these cases, a lack of facilities for identifying the bacterium, especially in countries where incidence is unknown, resulted in delays before diagnosis and definitive treatment. The classic resistance to colistin and gentamicin but sensitivity to amoxicillin/clavulanate seen in oxidase-positive, gram-negative rods may facilitate diagnosis.

In New Caledonia, the 4 patients had a variety of symptoms, from pneumonia, with or without septic shock, to cutaneous abscesses. The last case shows that exposure to *B. pseudomallei* does not always result in severe disease. The severity of illness probably depends on a balance between the bacterial strain's virulence, size of the infectious dose, delay before diagnosis, and immune status of the host (*2*). Risk factors for the patients were similar to those described elsewhere (*2**,**8*) and included diabetes, alcohol excess, chronic renal disease, and immunosuppression. Interestingly, all patients were heavy drinkers of kava, an extract of the plant *Piper methysticum*, which is drunk as an alternative to alcohol. Kava may be associated with melioidosis in some aboriginal communities in northern Australia (*10*). In New Caledonia, as in Australia, this "traditional" consumption is recent. Kava first appeared in New Caledonia ≈15 years ago as an import of Melanesian tradition mainly from Vanuatu. The roots are dried then pounded into powder and exported to New Caledonia and Australia where it is mixed with water to produce a brownish brew consumed for its psychoactive properties. Whether the association of melioidosis with kava consumption is an independent risk and whether it is because of possible ingestion of contaminated brew (*11*) or because of increased susceptibility of kava drinkers to melioidosis after exposure to *B. pseudomallei* requires further evaluation.

Melioidosis mainly affects persons who have direct contact with wet soil and have an underlying predisposition to infection. In the bush, many Melanesian persons spend a great deal of time outdoors with bare feet or wearing sandals, which increases percutaneous exposure to soil or muddy water during the wet season. New Caledonia often has periodic and heavy rain throughout the year. Four cases in 6 years represent an average annual incidence of 1.81/100,000 in the Northern Province, with 22.4/100,000 in the Ouegoa area, which suggests a high-prevalence focal region of melioidosis.

In conclusion, melioidosis cases have emerged in Melanesian persons, including those with diabetes, in a high-rainfall area in New Caledonia. Sampling of soil and ground water (and kava) for *B. pseudomallei* could be performed in this region to clarify the distribution of the bacterium and increase our understanding of this public health concern.
